# *Erianthus* germplasm collection in Thailand: genetic structure and phylogenetic aspects of tetraploid and hexaploid accessions

**DOI:** 10.1186/s12870-021-03418-3

**Published:** 2022-01-22

**Authors:** Shin-ichi Tsuruta, Suparat Srithawong, Suchirat Sakuanrungsirikul, Masumi Ebina, Makoto Kobayashi, Yoshifumi Terajima, Amarawan Tippayawat, Werapon Ponragdee

**Affiliations:** 1 Tropical Agriculture Research Front, Japan International Research Center for Agricultural Sciences (JIRCAS), Ishigaki, Okinawa, 907-0002 Japan; 2 Department of Agriculture, Khon Kaen Field Crops Research Center (KKFCRC), Khon Kaen, 40000 Thailand; 3 Present address: Biotechnology Research and Development Office (BIRDO), Department of Agriculture, Pathum Thani, 12110 Thailand; 4 Institute of Livestock and Grassland Science, National Agriculture and Food Research Organization (NARO), Nasushiobara, Tochigi, 329-2793 Japan; 5 Present address: Department of Agriculture, Faculty of Agriculture, Khon Kaen University, Khon Kaen, 40002 Thailand; 6 Present address: Field and Renewable Energy Crops Research Institute (FCRI), Department of Agriculture, Bangkok, 10900 Thailand

**Keywords:** *Erianthus arundinaceus*, *Saccharum*, Bioenergy, Germplasm, SSR, Genetic diversity, Chloroplast DNA

## Abstract

**Background:**

The genus *Erianthus*, which belongs to the “*Saccharum* complex”, includes C_4_ warm-season grasses. *Erianthus* species are widely distributed throughout Southeast Asia, East Asia and South Asia. *Erianthus arundinaceus* (Retz.) Jeswiet is highly adaptable to the environment, has a high percentage of dry matter, and is highly productive. Recently, this species has attracted attention as a novel bioenergy crop and as a breeding material for sugarcane improvement. Such interest in *E. arundinaceus* has accelerated the collection and conservation of its genetic resources, mainly in Asian countries, and also evaluation of morphological, agricultural, and cytogenetic features in germplasm collections. In Thailand, genetic resources of *E. arundinaceus* have been collected over the past 20 years and their phenotypic traits have been evaluated. However, the genetic differences and relatedness of the germplasms are not fully understood.

**Results:**

A set of 41 primer pairs for nuclear simple sequence repeats (SSRs) developed from *E. arundinaceus* were used to assess the genetic diversity of 121 *Erianthus* germplasms collected in Thailand; of these primer pairs, 28 detected a total of 316 alleles. A Bayesian clustering approach with these alleles classified the accessions into four main groups, generally corresponding to the previous classification based on phenotypic analysis. The results of principal coordinate analysis and phylogenetic analysis of the 121 accessions on the basis of the SSR markers showed the same trend as Bayesian clustering, whereas sequence variations of three non-coding regions of chloroplast DNA revealed eight haplotypes among the accessions. The analysis of genetic structure and phylogenetic relationships, however, found some accessions whose classification contradicted the results of previous phenotypic classification.

**Conclusions:**

The molecular approach used in this study characterized the genetic diversity and relatedness of *Erianthus* germplasms collected across Thailand. This knowledge would allow efficient maintenance and conservation of the genetic resources of this grass and would help to use *Erianthus* species as breeding materials for development of novel bioenergy crops and sugarcane improvement.

**Supplementary Information:**

The online version contains supplementary material available at 10.1186/s12870-021-03418-3.

## Background

According to the Fifth Assessment Report (AR5: 2013–2014) of the Intergovernmental Panel on Climate Change (IPCC), since the middle of the twentieth century, the natural environment has been exposed to severe global warming caused by human activities, and urgent mitigation efforts are needed, including energy-saving measures and the introduction of renewable energy sources [1]. Global warming will unavoidably harm food production, ecosystems, and water resources, and advance preparation is necessary to adapt to climate change [1]. Global climate change may affect stable agricultural production by causing droughts, floods, high-temperature injury, and emergence of novel pests [2, 3]. Thus, the establishment of sustainable agricultural production systems resilient to global climate change is one of the critical challenges that humans will face in the near future [1].

Diverse germplasm collections are essential for stable preservation and maintenance of ecotypes that might adapt to climate changes; these collections could provide indispensable materials for the development of novel cultivars with such adaptations. The genus *Erianthus*, which belongs to the “*Saccharum* complex”, includes C_4_ warm-season grasses. The complex includes five interbreeding genera—*Saccharum* L. (sugarcane), *Miscanthus* Andersson sect. *Diandra* Keng, *Erianthus* sect. *Ripidium*, Narenga Bor, and *Sclerostachya A. Camus*—that grow in a wide range of climatic conditions [4–6]. *Erianthus* sect. *Ripidium* has a basic chromosome number of *x* = 10 [7] and comprises seven closely related species: *E. elephantinus* Hook. f. (2*n* = 20), *E. hostii* Griseb. (2*n* = 20), *E. ravennae* (*L*.) P. Beauv. (2*n* = 20), *E. procerus* (Roxb.) Raizada (2*n* = 40), *E. kanashiroi* Ohwi (2*n* = 60), *E. arundinaceus* (Retz.) Jeswiet (2*n* = 30, 40, 60), and *E. bengalense* (Retz.) Bharadw. (2*n* = 20, 30, 40, 60) [7]. On the basis of the analysis of repulsion-phase linkage, Chen et al. [8] reported that *Erianthus* behaves like a true autopolyploid. Of the seven species, *E. arundinaceus* and *E. procerus* have recently attracted attention for their use in *Erianthus* and sugarcane breeding to increase biomass production [9, 10]. *Erianthus arundinaceus* is highly adaptable to the environment and is distributed in tropical and subtropical areas throughout Southeast Asia, East Asia, and South Asia. Accessions adapted to temperate zones [11] and high altitude [12] have also been found in East Asia. The plant height reaches more than 3 m [13, 14], and the high percentage of dry matter and high productivity of this species have attracted attention to *E. arundinaceus* as a novel bioenergy crop [11, 15]. Stress tolerance of this species in adverse environments also makes it a promising breeding material for the improvement of sugarcane [6, 10, 16, 17]. Such wide interest in *E. arundinaceus* has accelerated its collection and conservation, mainly in Asian countries, including India, China, and Thailand, which are rich in *Erianthus* genetic resources [9, 12, 18]. A number of *E. arundinaceus* accessions have also been collected in the temperate zone in Japan with the aim of exploring breeding materials for genetic improvement of its overwintering ability as a bioenergy crop candidate [19].

The collected genetic resources can be effectively used in breeding programs after multidimensional evaluation of their characteristics. In China [14] and Japan [11], morphological, agricultural, and cytogenetic features have been evaluated in *E. arundinaceus* germplasm collections. Genetic diversity assessment using molecular markers has been reported in *E. arundinaceus* accessions collected from India, China, the Philippines, Vietnam, Indonesia, and Japan for the purpose of bioenergy crop development and sugarcane improvement [20, 21]. Recently, the first cultivar of *E. arundinaceus*, ‘JES1’, was developed from a Japanese wild accession and was used as raw material for pellet fuel instead of wood pulp [11]. Intergeneric hybrids between *Saccharum* spp. and *E. arundinaceus* or between *E. procerus* and *S. officinarum* have been used to produce progeny by backcrossing for sugarcane improvement [17, 22, 23]. The genetic resources of *Erianthus* species from all over Thailand have been explored in the past 20 years, and two species, *E. arundinaceus* (2*n* = 40, 60) and *E. procerus*, have been found there. Thai *E. arundinaceus* accessions have been classified into Types I–III on the basis of phenotypic traits [9]. Types I and II are hexaploids (2*n* = 6*x* = 60). Type I has a hairy leaf sheath and can adapt to various environments such as open hill slopes and streambeds. Type II has a hairless leaf sheath covered with a waxy substance. This type has large buds capable of germinating and prominent root primordia, and is distributed mainly in the south of Thailand. Type III is a tetraploid (2*n* = 4*x* = 40) and is similar to Type II, but lacks wax on the leaf sheath. This type is found mainly along rivers and streams in Thailand. *Erianthus procerus* lacks hair and wax on the leaf sheath. This species has no prominent buds or root primordia, and is found in Thailand mainly on mountain slopes and on forest and field edges. There are no reports of the analysis of these genetic resources with molecular markers, and little is known about the genetic diversity of these *Erianthus* species in Thailand.

Molecular markers provide an objective evaluation of genetic variation in germplasms, which, being not influenced by environmental changes, are thus widely used to assess genetic diversity of plant genetic resources [24]. In the past decade, next generation sequencing (NGS) methodologies, which allow the genome-wide development of molecular markers and genotyping, have been applied to the polyploid species of warm-season grasses such as *Paspalum* [25], *Panicum* [26], and *Pennisetum* [27]. Using NGS data, we have developed simple sequence repeat (SSR or microsatellite) markers from the genomic DNA of hexaploid *E. arundinaceus* [28]. These SSR markers have been used successfully to estimate genetic diversity in *E. arundinaceus* collected in Japan and Indonesia [21]. In contrast to nuclear DNA markers, which are biparentally inherited, uniparentally inherited loci such as those in the chloroplast genome might provide information about the evolutionally history of germplasms [29, 30]. Therefore, a comparative analysis of both types of DNA polymorphisms could provide a more complementary and comprehensive insight into genetic diversification.

Genetic improvement depends on the diversity of available genetic resources; thus, it is essential to understand the genetic diversity of *E. arundinaceus* to facilitate breeding programs in this species. The main objective of the present research is to characterize the genetic variability in the *Erianthus* species of Thailand that has resulted in inter- and intraspecific variation in morphology. We investigated variability of SSR markers and partial chloroplast genome sequences of the *Erianthus* genetic resources of Thailand. On the basis of these data, we also analyzed the geographical distribution of the genetic diversity of *Erianthus* species in Thailand.

## Results

### SSR analysis

Among 41 SSR primer pairs developed from Japanese *E. arundinaceus* [28], 28 primer pairs resulted in scorable amplicons of the expected sizes in all accessions tested (*E. arundinaceus* and *E. procerus* included), with a total of 316 alleles (Tables S1 and S2). Allele number per locus ranged from 1 (ETR098) to 32 (ETR129), with an average of 11.3 (Table 1). Of the 28 SSRs, 27 (except ETR098) detected polymorphic loci.

**Table 1:** Characteristics of 28 SSR loci in 121 *Erianthus* accessions collected in Thailand

Locus		Expected size (bp) ^b^	Observed size range (bp)	Characteristics ^c^					
Name	Motif ^a^			*N*_A_	*N*_P_	*N*_G_	PIC	MI	*R*_p_
ED002	(CT)_8_	247	238–278	13	100.0	33	0.16	2.03	2.79
ED011	(AG)_8_	199	144–216	10	100.0	39	0.22	2.20	3.01
**ED035 **^d^	(AC)_8_	232	194–232	11	100.0	53	0.27	3.01	4.81
ED082	(AT)_8_	197	157–282	26	100.0	86	0.19	4.82	6.48
ED088	(AG)_10_	174	159–195	16	100.0	65	0.20	3.14	4.25
ED101	(GT)_8_	186	196–220	13	100.0	39	0.19	2.42	3.50
**ED106**	(CT)_11_	207	174–206	14	100.0	68	0.25	3.54	5.07
**ED113**	(AT)_8_	182	171–189	9	100.0	37	0.27	2.43	3.45
ED185	(AG)_12_	177	153–269	9	88.9	18	0.14	1.13	1.72
ED188	(GT)_9_	201	141–200	6	100.0	18	0.28	1.67	2.35
ED239	(CT)_8_	179	174–180	4	100.0	13	0.36	1.44	2.05
**ED265**	(GT)_8_	235	227–257	14	100.0	56	0.22	3.04	4.88
**ED307**	(GT)_8_	173	143–179	9	100.0	34	0.26	2.30	3.59
**ED316**	(GT)_8_	249	234–256	11	100.0	43	0.25	2.77	4.10
ETR044	(AGC)_7_	193	124–249	9	100.0	25	0.23	2.05	2.84
ETR047	(AGG)_6_	197	189–195	3	100.0	5	0.24	0.73	0.94
ETR074	(GGT)_5_	224	188–239	10	90.0	25	0.16	1.46	1.93
ETR077	(CCT)_5_	198	192–210	5	80.0	9	0.18	0.71	1.09
ETR083	(CTT)_14_	234	214–329	29	100.0	75	0.13	3.75	4.89
ETR097	(CCG)_6_	190	178–205	9	88.9	23	0.19	1.55	2.26
ETR098	(CTT)_5_	139	138	1	0.0	1	0.00	0.00	0.00
ETR104	(AGC)_5_	252	156–326	9	88.9	18	0.19	1.54	2.17
ETR107	(CCG)_5_	234	174–244	7	100.0	16	0.22	1.53	2.28
ETR124	(GAT)_7_	182	166–181	6	83.3	16	0.28	1.40	2.50
ETR129	(AAT)_7_	154	137–236	32	100.0	83	0.15	4.94	6.30
**ETR154**	(CCG)_6_	194	171–198	10	100.0	36	0.25	2.53	3.59
ETR169	(GTT)_10_	242	183–322	12	100.0	46	0.20	2.46	3.26
ETR172	(GCT)_5_	141	112–184	9	100.0	18	0.16	1.43	1.98
Maximum				32	100.0	86	0.36	4.94	6.48
Minimum				1	0.0	1	0.00	0.00	0.00
Mean (SD)				11.3 (7.2)	93.6 (19.2)	35.6 (23.5)	0.21 (0.07)	2.22 (1.15)	3.15 (1.55)

^a^ Motifs and numbers of repeat in *E. arundinaceus* accession ‘JW630’ used in SSR marker development

^b^ Size of PCR product in *E. arundinaceus* accession ‘JW630’ used in SSR marker development

^c^*N*_*A*_, numbers of amplified fragments; *N*_P_, % of polymorphic fragments; *N*_G_, numbers of genotypes; PIC, polymorphic information content; MI, marker index; *R*_p_, resolving power

^d^ Loci indicating high discriminatory power in all 121 accessions are shown in bold

To evaluate the informativeness of the 28 SSR primer pairs, we calculated polymorphic information content (PIC), marker index (MI), and resolving power (*R*_p_) for each locus in all accessions tested. These genetic parameters, as well as expected fragment sizes, observed size ranges, numbers of amplified fragments (*N*_A_), and percentage of polymorphic fragments (*N*_P_) and the numbers of genotypes (*N*_G_), are shown in Table 1 and S1. In all 121 accessions, PIC values ranged from 0.00 to 0.36, with an average of 0.21; MI ranged from 0.00 to 4.94, with an average of 2.22; and *R*_p_ ranged from 0.00 to 6.48, with an average of 3.15 (Table 1). In the 71 hexaploid *E. arundinaceus* accessions, the average values were 0.23 (range, 0.00–0.34) for PIC, 2.23 (0.00–5.82) for MI, and 3.19 (0.00–7.69) for *R*_p_ (Table S1). In the 16 tetraploid *E. arundinaceus* accessions, the average values were 0.24 (0.00–0.43) for PIC, 1.22 (0.00–4.77) for MI, and 1.88 (0.00–6.50) for *R*_p_ (Table S1). In the 34 *E. procerus* accessions, the average values were 0.25 (0.00–0.42) for PIC, 1.34 (0.00–2.93) for MI, and 2.09 (0.00–4.06) for *R*_p_ (Table S1). In all 121 accessions, the values of these parameters for 7 loci were larger than the average values of all loci, indicating their high discriminatory power (bold loci in Table 1).

### Genetic structure

**Fig. 1:**
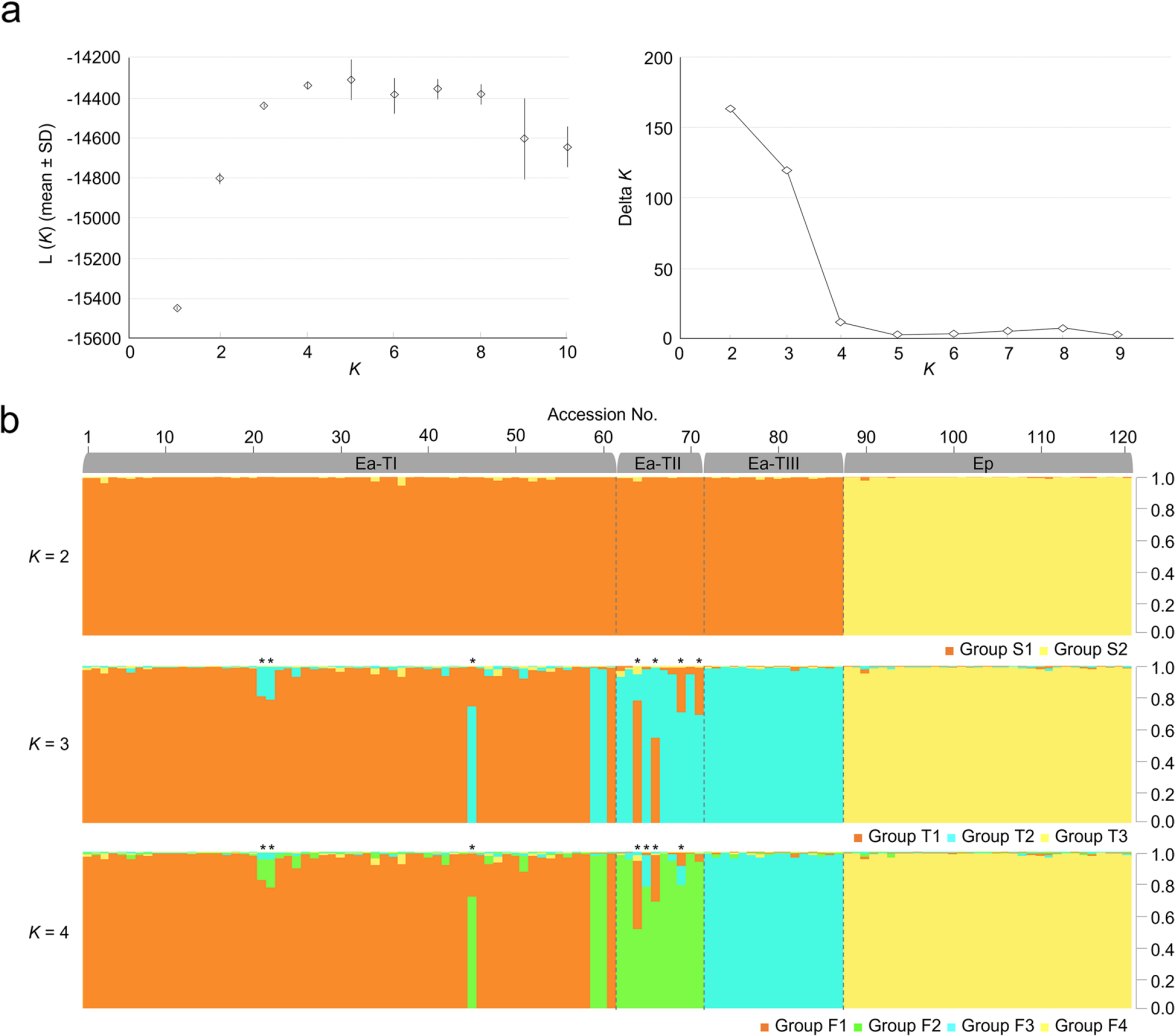
Genetic assignment of 121 *Erianthus* accessions using data from genotyping with 28 SSR primer pairs. **a** Estimation of the most likely number of groups using structure analysis: the mean values of log-likelihood for 10 independent runs for each value of *K* (left) and Δ*K* statistics for different *K* values based on the second-order rate of change in the log-likelihood function (right). **b** Bar plots of ancestry proportions for the Δ*K* values at *K* = 2, *K* = 3, and *K* = 4. Accessions identified as the admixture group are marked with asterisks. Ea-TI: *E. arundinaceus* Type I; Ea-TII: *E. arundinaceus* Type II; Ea-TIII: *E. arundinaceus* Type III; Ep: *E. procerus*

We used SSR genotyping data to determine the genetic structure of 121 *Erianthus* accessions. In the distribution of Δ*K* values, we found high values of Δ*K* at *K* = 2 and *K* = 3 (Fig. 1a). We assessed the individual proportion membership (qi) in the groups using the threshold value of 0.8, as used in other grass species; individuals with a value of ≥0.8 were considered to have a strong affinity to a group, and those with < 0.80 as an admixture [31–34]. With the threshold value of qi ≥0.80, the structure at *K* = 2 revealed two groups (S1 and S2; Fig. 1b, Table S2). Group S1 consisted of 87 *E. arundinaceus* accessions (71 hexaploids and 16 tetraploids). Group S2 consisted of 34 *E. procerus* accessions. At *K* = 3, three groups of accessions were defined (T1–T3; Fig. 1b, Table S2) with the same cutoff for membership assignment. Group T1 (54 accessions) included *E. arundinaceus* hexaploids. Group T2 included 24 *E. arundinaceus* accessions (8 hexaploids and 16 tetraploids). Group T3 included 34 *E. procerus* accessions. Seven hexaploid *E. arundinaceus* accessions—ThE01–012 (map No. 21), ThE01–013 (22), ThE02–091 (45), ThE02–086 (64), ThE10–003 (66), ThE10–008 (69), and ThE10–021 (71)—were identified as the admixture group; all accessions were an admixture of T1 and T2.

The structure at *K* = 4 revealed four groups (F1–F4; Fig. 1b, Table S2). Groups F1 (54 accessions) and F2 (8 accessions) included *E. arundinaceus* hexaploids. Group F3 included 16 tetraploid *E. arundinaceus* accessions. Group F4 included 34 *E. procerus* accessions. The remaining seven accessions of hexaploid *E. arundinaceus*—ThE01–012 (21), ThE01–013 (22), ThE02–091 (45), ThE02–086 (64), ThE02–087 (65), ThE10–003 (66), and ThE10–008 (69)—were identified as the admixture group; Nos. 65 and 69 were admixtures of F2 and F3, and the other accessions were admixtures of F1 and F2. The analysis also found two accessions, ThE10–004 (59) and ThE10–005 (60), with ambiguous phenotypic clustering.

### Phylogenetic relationships

**Fig. 2:**
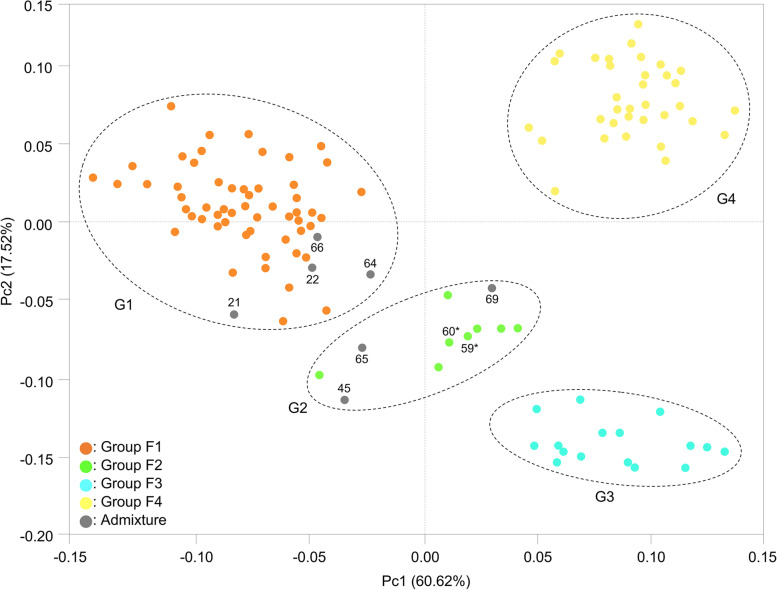
Principal coordinate analysis of 121 *Erianthus* accessions based on the Bruvo distance between individuals calculated in Polysat software. Colors indicate four groups corresponding to those in Fig. 1 at *K* = 4. The admixture group is indicated by grey circles. Map numbers of seven accessions assigned to the admixture group are shown next to the circles. Two accessions with ambiguous phenotypic clustering are marked with asterisks next to the map numbers. Dashed circles indicate grouping (G1–G4). Numbers on each axis indicate the proportion of variance explained by each principal coordinate

**Fig. 3:**
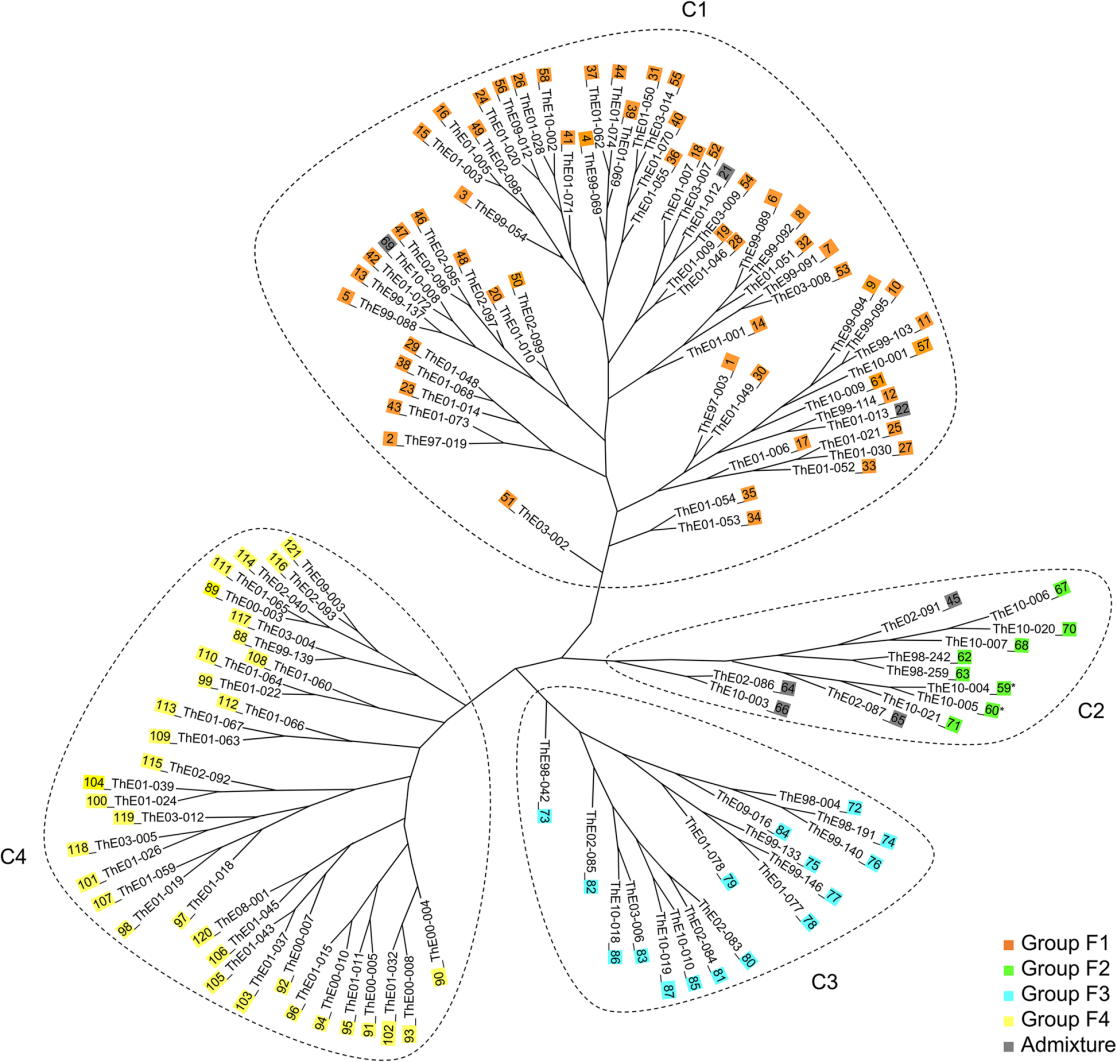
Unrooted neighbor-joining tree of 121 *Erianthus* accessions based on Nei’s minimum distance. The color of each accession corresponds to that in Fig. 1 at *K* = 4. Two accessions with ambiguous phenotypic clustering are marked with asterisks

To better understand the genetic structure and relationships among Thai *Erianthus* accessions, we performed a principal coordinate analysis (PCoA) and a phylogenetic analysis based on the genotyping data obtained from 28 SSRs. The results of PCoA revealed four main groups (G1–G4; Fig. 2, Table S2). The admixture group overlapped with groups G1 and G2 (Fig. 2, Table S2). Phylogenetic analysis of the 121 accessions using Nei’s minimum distance on the basis of 316 alleles detected with the 28 SSR loci showed four major groups (C1–C4; Fig. 3), supporting the population structure determined by structure analysis. Groups C1 (59 accessions) and C2 (12 accessions) included hexaploid *E. arundinaceus*. Group C3 contained 16 tetraploid *E. arundinaceus* accessions. Group C4 contained 34 *E. procerus* accessions. The grouping of two accessions—ThE10–004 (59) and ThE10–005 (60)—based on phenotypic variation [9] was inconsistent with that based on genetic variation (Figs. 2 and 3; Table S2). On the basis of phenotypic variations, these accessions have been classed into Type I [9], but our PCoA and phylogenetic analysis classed them into groups G2 and C2, respectively, which consisted mostly of Type II accessions.

### Genetic diversity and differentiation among groups

To evaluate genetic diversity and differentiation in groups based on polymorphic SSRs, we calculated the genetic diversity parameters for the groups defined at *K* = 4, which could explain the genetic characteristics of Thai *Erianthus* species in more detail. In this analysis (Table 2), we excluded the seven accessions identified as the admixture group. *N*_A_ ranged from 5.54 in group F3 to 10.14 in F1, with an average of 7.01. The effective number of alleles (*N*_Ae_) ranged from 3.97 (F3) to 5.13 (F1), with an average of 4.51. Allelic richness (*A*_R_) ranged from 4.47 (F3) to 5.76 (F1), with an average of 5.04. Gene diversity (*H*_e_) ranged from 0.72 (F3) to 0.78 (F1), with an average of 0.75. The values of the inbreeding coefficient (*F*_i_) were negative in all groups and ranged from − 0.27 (F1) to − 0.39 (F3), with an average of − 0.34. Group F1 had the highest values of all parameters except *F*_i_.

**Table 2:** Statistical analysis of genetic diversity of each group

Group ^a^	*N *^b^	Genetic diversity parameters ^c^				
		*N*_A_	*N*_Ae_	*A*_R_	*H*_e_	*F*_i_
F1	56	10.14	5.13	5.76	0.78	−0.27
F2	8	6.11	4.77	5.19	0.76	−0.34
F3	16	5.54	3.97	4.47	0.72	−0.39
F4	34	6.43	4.15	4.72	0.74	−0.34

^a^ Groups assigned by structure analysis (*K* = 4). The five accessions assigned to the admixture group at the 0.80 cutoff value were excluded

^b^ Number of accessions

^c^*N*_A_, number of amplified fragments; *N*_Ae_, effective number of alleles; *A*_R_, allelic richness; *H*_e_, gene diversity; *F*_i_, inbreeding coefficient

To estimate the degree of relative genetic differentiation among the groups, we calculated population differentiation (*F*_st_) among all groups (Table 3). *F*_st_ ranged from 0.03 to 0.05 (average 0.04). The effective migration rate (*N*_m_) ranged from 4.88 between groups F3 and F4 to 8.22 between F1 and F4 (Table 3), with an average of 6.58.

**Table 3:** Pairwise *F*_st_ (above diagonal) and *N*_*m*_ values (below diagonal) among the groups

	F1 ^a^	F2	F3	F4
F1		0.031	0.039	0.030
F2	8.143		0.039	0.047
F3	6.410	6.494		0.051
F4	8.224	5.319	4.883	

^a^ Groups assigned by structure analysis (*K* = 4). The five accessions assigned to the admixture group at the 0.80 cutoff value were excluded

### Geographical distribution of genetic diversity

**Fig. 4:**
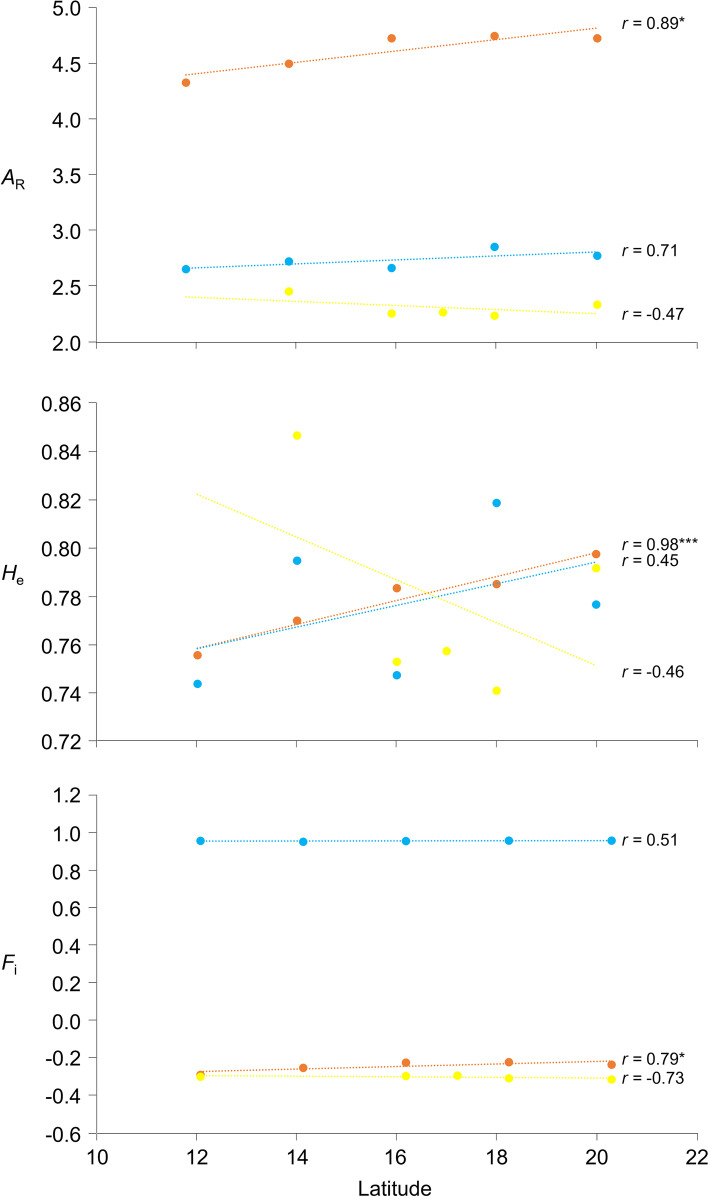
Correlations between genetic diversity parameters (*A*_R_, *H*_e_, and *F*_*i*_) and latitude. The analysis was performed for hexaploid *E. arundinaceus* (orange), tetraploid *E. arundinaceus* (blue), and *E. procerus* (yellow). Latitudes were rounded to integers. Significant correlations are indicated (*p < 0.01 and ***p < 0.0001)

To evaluate the relationships between genetic diversity and geographical distribution, we investigated the correlation between genetic distance and geographic distance among accessions using Mantel’s test. The correlation was significant for the 71 hexaploid *E. arundinaceus* accessions (*r* = 0.369, *p* = 0.0001), but not for the 16 tetraploid *E. arundinaceus* accessions (*r* = 0.135, *p* = 0.1112) or the 34 *E. procerus* accessions (*r* = 0.154, *p* = 0.0118), when *p* < 0.001 was considered statistically significant. In addition, we determined the correlations between genetic diversity parameters (*A*_R_, *H*_e_, and *F*_i_) and geographic distribution (Fig. 4). *A*_R_, *H*_e_, and *F*_i_ for hexaploid *E. arundinaceus* were positively correlated with latitude (*r* = 0.89*, 0.98*** and 0.79*, respectively). In tetraploid accessions (*E. arundinaceus* and *E. procerus*), no significant correlations were found. These values were not significantly correlated with longitude in either tetraploid or hexaploid *Erianthus* (data not shown).

### Chloroplast haplotype variation

We also compared sequences of three non-coding regions (*rps*16–*trn*Q, *atp*A–*rps*14, and *rpl*16–*rps*3) of chloroplast DNA (cpDNA) among the 121 *Erianthus* accessions. The lengths of these regions were 747, 810–839, and 529–544 bp, respectively, and the lengths of concatenated sequences were 2093–2126 bp (Table S3). The aligned sequences of these regions were 747, 846 and 544 bp, respectively, and the concatenated alignment of the three regions was 2126 bp (Tables S3, S4). We identified 11 variations in the sequence, of which 10 were parsimony-informative sites. On the basis of the concatenated sequences, haplotypes H1–H8 were identified (Table S4). Haplotype diversity, nucleotide diversity, and neutrality were estimated in the absence of indels for each group identified in the structure analysis (*K* = 4) and are summarized in Table 4. Overall haplotype diversity (*h*) was 0.78. Haplotypes H1–H7 were identified in group F1, which had the highest value of haplotype diversity (*h* = 0.78). The overall values of nucleotide diversity parameters were 1.23 for *Pi*, 1.68 for *θ*, and 0.81 for π. The highest values of *Pi* (1.36), *θ* (1.74), and π (0.084) were found in group F1. The neutrality test statistics (*D*_Fu and Li_, *F*_Fu and Li_, *F*_Fu_, and *D*_Tajima_) were not significant in any group and did not reveal a deviation from neutrality in the regions examined in all 121 accessions.

**Table 4:** Statistical analysis of haplotype diversity, nucleotide diversity, and neutrality for each group using the aligned sequences of three cpDNA regions

Group ^a^	*N *^b^	Haplotype diversity		Nucleotide diversity			Neutrality tests			
		Haplotypes ^c^	*h*	*Pi* (10^−3^)	*θ*	π (10^− 3^)	*D*_Fu and Li_	*F*_Fu and Li_	*F*_Fu_	*D*_Tajima_
F1	56	H1(20), H2(5), H3(16), H4(5), H5(4), H6(1), H7(5)	0.78	1.36	1.74	0.84	1.29	1.67	1.84	1.68
F2	8	H4(8)	–	–	–	–	–	–	–	–
F3	16	H1(8), H7(8)	0.53	0.25	0.30	0.14	0.69	1.03	1.36	1.53
F4	34	H1(21), H5(7), H6(5), H8(1)	0.57	0.86	1.22	0.58	0.23	0.63	2.28	1.26
AM	7	H3(2), H4(4), H7(1)	–	–	–	–	–	–	–	–
Overall	121	H1(49), H2(5), H3(18), H4(17), H5(11), H6(6), H7(14), H8(1)	0.78	1.23	1.68	0.81	0.53	0.94	1.83	1.27

^a^ Groups assigned by structure analysis (*K* = 4). AM, accessions assigned to admixture at the 0.80 cutoff

^b^ Number of accessions in each group

^c^ Number of accessions in each haplotype is shown in parentheses

**Fig. 5:**
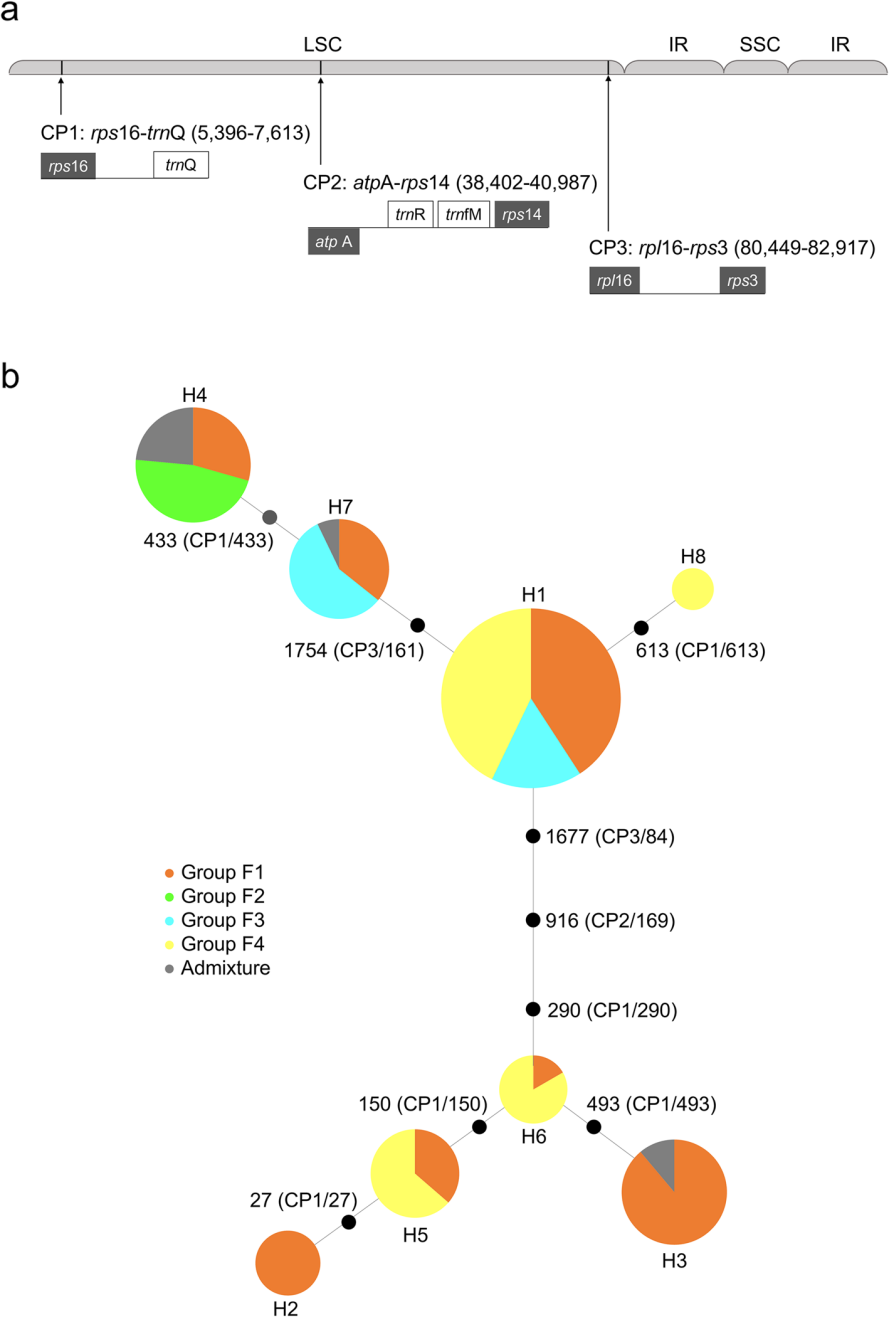
Median-joining network of eight haplotypes (H1–H8) based on sequence variations in three non-coding regions of *E. arundinaceus* cpDNA. **a** Locations of the three regions (*rps*16–*trn*Q, *atp*A–*rps*14, and *rpl*16–*rps*3). The position (bp) of each region in full-length cpDNA of *E. arundinaceus* accession ‘JW630’ is given in parentheses. LSC, large single-copy region; IR, inverted repeat; SSC, small single-copy region. **b** Circle sizes are proportional to haplotype frequency, and their colors correspond to those in Fig. 1 at *K* = 4. Positions of mutational steps in concatenated sequences between haplotypes are shown next to the branches. Positions of mutational steps in each cpDNA region are shown in parentheses. Haplotype of each accession is indicated in Table S2

A parsimonious network among haplotypes of the 121 accessions was generated from the sequence dataset of the three chloroplast non-coding regions (Fig. 5). The haplotypes in the network were clearly split into the upper clade (H1, H4, H7, and H8) and lower clade (H2, H3, H5, and H6), and the connection among lineages extended to three steps. Haplotype H1 was predominant (56 of the 121 accessions, 46.3%). In the upper clade, H7 and H8 were generated from H1 by one mutational step, and H4 was generated from H7 by one additional mutational step. In the lower clade, H6, connected to the upper clade, was generated from H1 via three mutational steps. H3 and H5 were separated from H6 by one mutational step, and H2 was generated from H5 by one additional mutational step.

## Discussion

The development of a new cultivar is influenced by the distribution of diversity in available genetic resources. There are some reports of genetic diversity assessment of the *Erianthus* genetic resources. Here, we characterized previously unknown genetic properties of *Erianthus* species in Thailand at the molecular level.

Two *Erianthus* species, *E. arundinaceus* and *E. procerus*, with different phenotypic characteristics and ploidy, have been identified in Thailand [9]. We used 41 SSR primer pairs developed from the nuclear genome of a Japanese *E. arundinaceus* accession [28] to genotype the Thai *Erianthus* collection. Of these primer pairs, 28 amplified products of the expected sizes in all 121 *E. arundinaceus* and *E. procerus* accessions, suggesting the usefulness of these primer pairs in genetic diversity analysis of *Erianthus* species in Thailand.

### Genetic structure of Thai *Erianthus*

Structure analysis based on genotyping data from the 28 SSR primers indicated high values of Δ*K* at *K* = 2 and *K* = 3. The structure at *K* = 2 revealed two groups (S1 and S2; Fig. 1, Table S2), corresponding to the two *Erianthus* species. At *K* = 3, three groups of accessions were defined (T1–T3; Fig. 1, Table S2). Of the three structure groups, one included mainly hexaploid *E. arundinaceus*, and the other two included tetraploid species (*E. arundinaceus* and *E. procerus*). In this analysis, seven accessions showed admixed ancestry between tetraploid and hexaploid *Erianthus* (Table S2). However, no traces of mating between accessions with different ploidy were identified from the fragment patterns. Using cytogenetic analysis, Tagane et al. [9] have reported that the *E. arundinaceus* accessions identified as admixed in this study were hexaploids. Another population structure was indicated by Δ*K* at *K* = 4. This structure could explain genetic characteristics of Thai *Erianthus* species in more detail. Tetraploid and hexaploid *E. arundinaceus* accessions, which were identified as the same group (T2) at *K* = 3, were defined as different groups (F2 and F3) at *K* = 4, except ThE02–087 (65). At *K* = 4, eight hexaploid accessions (Nos. 59, 60, 62, 63, 67, 68, 70, and 71) were defined as a group distinct from the 16 tetraploid *E. arundinaceus* accessions, although they belonged to the same group as tetraploid *E. arundinaceus* or to the admixture group at *K* = 3. The results of PCoA and phylogenetic analysis of the 121 accessions showed the same trend as the structure analysis at *K* = 4. On the basis of phenotypic analysis, Tagane et al. [9] divided Thai *E. arundinaceus* into Types I and II (2*n* = 6*x* = 60), and Type III (2*n* = 4*x* = 40). Our results of grouping at *K* = 4 are generally consistent with those results, except for seven accessions (Nos. 21, 22, 59, 60, 65, 66, and 69). We used the grouping at *K* = 4 in subsequent analysis.

### Genetic differentiation among groups

Genetic differentiation among all pairs of groups from the structure analysis (*K* = 4) was estimated using *F*_st_. At *F*_st_ > 0.15, genetic differentiation between populations is considered to be significant [35]. The genetic differentiation among the groups was low, as the *F*_st_ values were < 0.15 (0.030–0.051).

The analysis of genetic structure and phylogenetic relationships found some accessions that were genetically admixed (Nos. 21, 22, 45, 64, 65, 66, and 69) and some with ambiguous phenotypic clustering (Nos. 59 and 60). These results suggest natural hybridization events. Tagane et al. [9] plotted ThE02–091 (No. 45 in this study) and ThE02–086 (No. 64) in the intermediate regions in principal component analysis and canonical discriminant analysis based on phenotypic variations, and assumed these accessions to be hybrids between hexaploid (Type I) and tetraploid *E. arundinaceus* (Type III). Because flowering time differs among the three types of Thai *E. arundinaceus* [9], intercrossing among them seems to be difficult. This is one of the factors causing genetic differentiation in populations, and it could prevent genetic diversification based on hybridization events. In relation to gene flow, *N*_m_ between groups (4.88–8.22) indicated that the possibility of random mating between the populations was high (*N*_m_ > 4 [36, 37];), suggesting that gene flow has occurred among some accessions and thus may have led to a low genetic differentiation between groups. The flowering periods of the hexaploid *E. arundinaceus* Types I and II overlap slightly [9]. Because some accessions with contradictory phenotypic and genetic groupings were collected at locations geographically close to each other, the possibility that they have arisen by clonal propagation or genetic exchange between the types should not be completely dismissed. Further analysis with a greater number of DNA markers could clarify the origin of hybridity in these accessions.

### Genetic diversity and distribution

The SSR markers used in this study indicated a high degree of genetic diversity in group F1, which consisted of hexaploid *E. arundinaceus*. The basic chromosome number in *E. arundinaceus* is *x* = 10, and individuals with 2*n* = 30, 40, or 60 have been found [7, 38]. Type I *E. arundinaceus* is hexaploid and, among all regions, is found mainly in Thailand. Altered ploidy is one of the factors affecting genetic diversity [39, 40]. Among tetraploids, the mean values of genetic variation as indicated by Nei’s similarity index were 0.69 (range 0.07–0.86) in *E. arundinaceus* and 0.75 (range 0.50–0.96) in *E. procerus*, and were lower than in the hexaploid accessions (mean 0.86, range 0.04–0.96; data not shown). Zhang et al. [41] suggested that physical isolation by geographical barriers such as oceans, mountains, and rivers might decrease the level of genetic variation in *Erianthus* germplasms collected across China, including in an island area, because limited gene flow from outside reduces genetic diversity. Such effect of isolation on genetic diversity can also be inferred from the significant correlation between genetic distance and physical distance among the hexaploid *E. arundinaceus* accessions in this study. The diversity of accessions from Indonesia is lower than that in other Asian countries [20], as suggested by amplified fragment length polymorphism (AFLP) analysis. Correlation between genetic diversity and geographical distribution in this study showed that the degree of genetic diversity in hexaploid *E. arundinaceus* tended to increase with latitude. From phenotypic differences, it was suggested that genetic divergence of *E. arundinaceus* in Thailand could have occurred through adaptive evolution to different natural habitats [9]. These ideas together with our results suggest that not only isolation by geographical barriers but also adaptive radiation and parallel or convergent evolution accompanying changes in ecological conditions could affect genetic diversity in Thai *Erianthus* germplasms. The assessment of genetic diversity among germplasms collected over a wider area could improve our understanding of the geographical distribution of genetic diversity in *Erianthus* species and provide novel insights into the genetic basis of environmental adaptability of these species.

Information on the evolutionary history would also help us to understand the establishment of the current genetic diversity of *Erianthus* in Thailand. Type I hexaploid *E. arundinaceus* and tetraploid *E. procerus* were clustered into different groups in the structure and phylogenetic analyses. However, the degree of genetic divergence between these two groups was not high in comparison with that among other groups (Table 3). Because the chloroplast genome is maternally inherited in most angiosperm species, its diversity provides insight into the maternal evolutionary history and relationships among species with different chromosome numbers. Fu and Allaby [42] have reported that *Linum* species with different chromosome numbers share sequence variations in their chloroplast genomes. In this study, the network analysis based on variations in partial cpDNA sequences detected 8 haplotypes (Table 5), of which 5 (except H2, H3, and H8) were detected across the phenotypic types (Types I–III) and species (*E. arundinaceus* and *E. procerus*), even if the accessions had different chromosome numbers. For example, the tetraploid *E. procerus* tended to share sequence variants with hexaploid *E. arundinaceus* of Type I, suggesting that the current hexaploid *E. arundinaceus* and *E. procerus* could have diverged from a common matrilineal ancestor relatively recently. Therefore, the low degree of genetic divergence between groups detected in this study is not surprising. Similar results have been reported by Tagane et al. [9], who showed that these species are phenotypically similar and overlap in principal component analysis of phenotypic traits. A more detailed analysis of cpDNA polymorphisms could provide information useful for understanding the evolutionary relationships between and within *Erianthus* species.

**Table 5:** *Erianthus* accessions used in this study

Species	No. of accessions	No. of chromosomes	Ploidy	Phenotype ^a^	Map Nos. ^b^
*E. arundinaceus*	61	60	Hexaploid	Type I	1–61
*E. arundinaceus*	10	60	Hexaploid	Type II	62–71
*E. arundinaceus*	16	40	Tetraploid	Type III	72–87
*E. procerus*	34	40	Tetraploid	*E. procerus*	88–121

^a^ Classification based on phenotypic traits [9]

^b^ Accession numbers correspond to those in Table S2 and Fig. S1

Genetic resources of *Erianthus* species are distributed extensively in Southeast Asia, East Asia, and South Asia. Accessions adapted to temperate zones and high altitudes have also been explored and collected, mainly in China and Japan [11, 12]. A previous study in *Erianthus* species suggested that the degree of genetic diversity or similarity among populations and individuals could be influenced by genetic isolation due to geographical barriers [41]. We did not address the degree of genetic diversity between genetic resources from Thailand and other countries, and further investigations will be needed. In particular, a comprehensive analysis with common DNA markers targeting *Erianthus* accessions collected in Asian countries, where the genetic resources are plentiful, would not only clarify the position of the Thai germplasms, but also provide information on genetic characteristics of the available resources of *Erianthus* species. This knowledge would allow efficient maintenance and conservation of the genetic resources of these species of large grasses and would facilitate the use of *Erianthus* species as breeding materials for the development of novel bioenergy crops and the improvement of sugarcane, which is now in progress in some countries [11, 15, 43, 44]. Such studies might be an important step toward mitigation of and adaptation to global warming.

## Conclusions

In this study, we characterized the genetic diversity of *Erianthus* germplasms collected across Thailand by using SSR markers developed from genomic DNA of Japanese *E. arundinaceus*. These markers are highly polymorphic in Thai *Erianthus* accessions and could be useful for evaluation of genetic resources in *Erianthus* species. Thai *Erianthus* accessions were classified into four groups, generally corresponding to the previous classification based on phenotypic analysis. Some *E. arundinaceus* accessions were regarded as intraspecific hybrids. These results reveal the genetic basis for the Thai *Erianthus* germplasm collection diversity and provide useful resources for genetic study and breeding in *Erianthus* species.

## Methods

### Plant materials and DNA extraction

We used 71 hexaploid and 16 tetraploid *E. arundinaceus* accessions and 34 tetraploid *E*. *procerus* accessions (121 accessions in total; Table 5 and S2). All accessions were wild *Erianthus* collected across Thailand since 1997 in the framework of the collaborative research project between DOA (Development of Agriculture, Ministry of Agriculture and Cooperatives, Thailand) and JIRCAS (Japan International Research Center for Agricultural Sciences, Japan). The accessions were classified on the basis of morphological characters [13, 45]; they are maintained in the Tha Phra field at the Khon Kaen Field Crop Research Center, DOA, Khon Kaen, Thailand. On the basis of the coordinate data, the geographic distribution of these accessions was visualized in DIVA-GIS v. 7.5 software [46] (Fig. S1).

Genomic DNA was extracted from approximately 100 mg of freshly harvested leaves of each accession using a DNeasy Plant Mini Kit (Qiagen, Hilden, Germany). Its quality was assessed by agarose gel electrophoresis, and its concentration was determined with a NanoDrop 1000 spectrophotometer (Thermo Fisher Scientific Inc., Waltham, MA, USA) and adjusted to 10 ng/μL.

### Amplification of SSRs

The 41 SSR primer pairs developed from the wild *E. arundinaceus* accession ‘JW630’, collected in Japan [28], were used for PCR amplification of Thai *Erianthus* accessions. Of these primer pairs, 28 showed high stability and high reproducibility and were used to genotype the accessions (Table S2). PCR based on the M13-tailed primer method [47] was performed as described [28]. PCR products amplified with universal M13 primers labeled with each fluorophore (FAM, HEX, or NED) were diluted 1:5 and aliquots were combined with 12 μL deionized formamide (Amresco, Solon, OH, USA) and 0.25 μL ROX500 size standard (Life Technologies, Carlsbad, CA, USA). This mixture was heated at 98 °C for 2 min and then chilled on ice to denature the DNA before loading. Alleles were separated in an ABI3100 Genetic Analyzer (Life Technologies), and the allele sizes were determined automatically using GeneMapper v. 5.0 software (Life Technologies). The data included peaks that may be caused by artefacts often encountered in microsatellite analysis, such as stutter, peak broadening and shift. To minimize genotyping error, the allele sizes were checked and edited manually, and some samples were re-analyzed to confirm ambiguous genotypes.

Each fragment was considered as a dominant marker and was scored as 1 (presence) or 0 (absence), because the *E. arundinaceus* accessions used in this study are polyploid. The binary genotypic data were used to assess the discriminatory power and utility of each locus on the basis of PIC [48], MI [49, 50], *R*_p_ [51], expected fragment sizes, observed size range, *N*_A_, *N*_P_, and *N*_G_.

### Amplification of chloroplast non-coding regions

Three non-coding intergenic spacer regions of cpDNA—*rps*16–*trn*Q, *atp*A–*rps*14, and *rpl*16–*rps*3—were sequenced in all *Erianthus* accessions by using Sanger sequencing of each PCR product. Sequence variations in these regions were identified between *E. arundinaceus* accessions from Japan and Indonesia [52]. Primer pairs to amplify each region (Table S5) were designed from the chloroplast genome sequence of ‘JW630’ (GenBank accession No. LC160130). PCR was performed in a 15-μL mixture containing genomic DNA (20 ng), 5× PrimeSTAR buffer (TaKaRa, Shiga, Japan), 0.4 mM each dNTP (TaKaRa), 5 pmol of each specific forward and reverse primer, and 0.5 units PrimeSTAR HS-DNA polymerase (TaKaRa) in a GeneAmp PCR System 9700 thermal cycler (Life Technologies) as follows: 98 °C for 1 min, followed by 30 cycles of 98 °C for 15 s, 56 °C for 15 s, and 72 °C for 2.5 min. Amplification products were purified with a QuickStep2 PCR Purification Kit (Edge Biosystems, Gaithersburg, MD, USA) and were used as templates for sequencing. Cycle-sequencing was performed with a BigDye Terminator Cycle Sequence Kit v. 3.1 (Life Technologies) using specific primers (Table S5) in the same thermal cycler. Sequencing products were purified on a Sephadex G-50 column (GE Healthcare, Uppsala, Sweden) and sequenced in an ABI3500 genetic analyzer (Life Technologies).

### Data analysis

SSR profiles of 121 accessions were used to investigate the population genetic structure through model-based Bayesian clustering analysis in STRUCTURE v. 2.3.4 software [53, 54]. The data for tetraploids were treated as for hexaploids (i.e., the last two of six rows per individual in tetraploids were coded as missing data) to enable simultaneous analysis of the mixed-ploidy data. Recessive alleles were considered to be present (recessive alleles = 1), as described in the recessive allele approach for polyploid species [55]. The population number (*K* = 1–10) was tested in an admixture ancestry model with correlated allele frequencies. Each run was performed in 10 replicates for each *K* value, with a burn-in period of 100,000 steps followed by 100,000 Markov Chain Monte Carlo (MCMC) iterations. The optimal *K* values were determined using the ad hoc statistic Δ*K*, which was estimated as the rate of change in the log probability of data between successive *K* values [56] in the online application STRUCTURE HARVESTER [57]. The optimal alignment for 10 replicate runs was determined with the full search algorithm in CLUMPP 1.1.2 software [58], and then the inferred clusters were visualized as color bar plots in DISTRUCT 1.1 software [59].

Structural features were assessed by PCoA and phylogenetic tree analysis using SSR genotyping data. PCoA based on Bruvo distances [60] among individuals was performed in Polysat v. 1.4 in the R statistical software package. We also calculated pairwise Nei’s minimum distance among all accessions and constructed a phylogenetic tree of the 121 accessions by using the neighbor-joining method [61] based on Nei’s similarity index. The analysis was conducted in Populations 1.2.32 [62] and the tree was visualized in Figtree v. 1.4.2 software [63].

On the basis of SSR genotyping data, genetic diversity and allele frequencies in each group were estimated through the *N*_A_, *N*_Ae_, *A*_R_, *H*_e_, and *F*_i_ statistics. The coefficients of genetic differentiation between each group assigned by the structure analysis were estimated with the parameter *F*_st_ from the analysis of molecular variance (AMOVA); *F*_st_ / (1 − *F*_st_) was used. Statistical analyses were implemented in SPAGeDi 1.5 software [64], which was designed to analyze data from polyploid species. *N*_*m*_ between groups was estimated from *F*_st_, taking into account sample size and the number of loci [65, 66], as *N*_m_ = (1 − *F*_st_) / 4*F*_st_ [67].

A matrix of linear distances (km) among all genotypes was constructed on the basis of the geographic coordinates of the accessions in Geographic Distance Matrix Generator v. 1.2.3 software [68]. This matrix was compared to the genetic distance matrix (Nei’s minimum distance based on SSR genotyping) using Mantel’s correlation test [69] based on 1000 random permutations in GenoDive v. 2.0b27 software [70]. The correlation coefficients between genetic diversity parameters (*A*_R_, *H*_e_, and *F*_i_) and latitude and longitude were calculated in JMP 8.0 software (SAS Institute, Cary, NC, USA).

Sequences of the three chloroplast non-coding regions were concatenated and aligned in BioEdit 7.2.5 software [71]. After manual editing, haplotype and nucleotide diversities were estimated in each group using *h* [72], *Pi* [72], Watterson’s θ [73], and Tajima’s π [74] statistics. Tests of neutral evolution were performed as described [75–77]. All parameters were calculated in DnaSP v. 5.10.01 software [78]. Indels in the alignment were treated as missing data. The phylogenetic network of the inferred haplotypes was also constructed using concatenated alignments and a maximum-parsimony method based on a median-joining algorithm [79] in Network 4.6 software (http://www.fluxus-engineering.com/).

## Supplementary Information

**Additional file 1: Table S1**. Characteristics of 28 SSR loci in 121 Thai *Erianthus* accessions.

**Additional file 2: Table S2**. Sampling locations of 121 *Erianthus* accessions collected in Thailand, their grouping by each analysis, and size of amplified fragment in each accession.

**Additional file 3: Table S3**. GenBank accession numbers of three non-coding regions of chloroplast DNA and their concatenated sequences in 121 Thai *Erianthus* accessions.

**Additional file 4: Table S4**. Statistical analysis of haplotype diversity, nucleotide diversity, and neutrality in each chloroplast DNA region in 121 Thai *Erianthus* accessions.

**Additional file 5: Table S5**. Sequences of primers used for amplification and sequencing of chloroplast DNA regions in Thai *Erianthus* accessions.

**Additional file 6: Figure S1**. Geographic locations of 121 *Erianthus* accessions collected in Thailand, and a pie chart of the populations and a bar chart of ancestry proportion in the 7 admixtures. Colors correspond to those in Fig. 1 at *K* = 4. Admixture group is indicated in gray. Accession numbers are listed as map No. in Table S2.

## Data Availability

All data generated and analyzed during this study, including primer sequences and grouping of the accessions, are included in this published article and its supplementary information files. All cpDNA sequences generated in this study are available in the NCBI database (https://www.ncbi.nlm.nih.gov/) with accession numbers LC636829–LC637191 (Table S3).

## Abbreviations

AFLP: Amplified fragment length polymorphism
AMOVA: Analysis of molecular variance
*A*_R_: Allelic richness
cpDNA: Chloroplast DNA
*F*_i_: inbreeding coefficient
*F*_st_: population differentiation
*H*_e_: gene diversity
MI: Marker index
*N*_A_: Number of amplified fragments
*N*_Ae_: effective number of alleles
NGS: Next generation sequencing
*N*_m_: Effective migration rate
*N*_G_: Number of genotypes
*N*_P_: Percentage of polymorphic fragments
PCoA: Principal coordinate analysis
PCR: Polymerase chain reaction
PIC: Polymorphic information content
*R*_p_: Resolving power
SSR: Simple sequence repeat
