# Supplementary figures and images for: Erianthus germplasm collection in Thailand: genetic structure and phylogenetic aspects of tetraploid and hexaploid accessions
Source: BMC Plant Biol. 2022 Jan 22;22:45. doi: 10.1186/s12870-021-03418-3 (PMC8783461; doi:10.1186/s12870-021-03418-3)

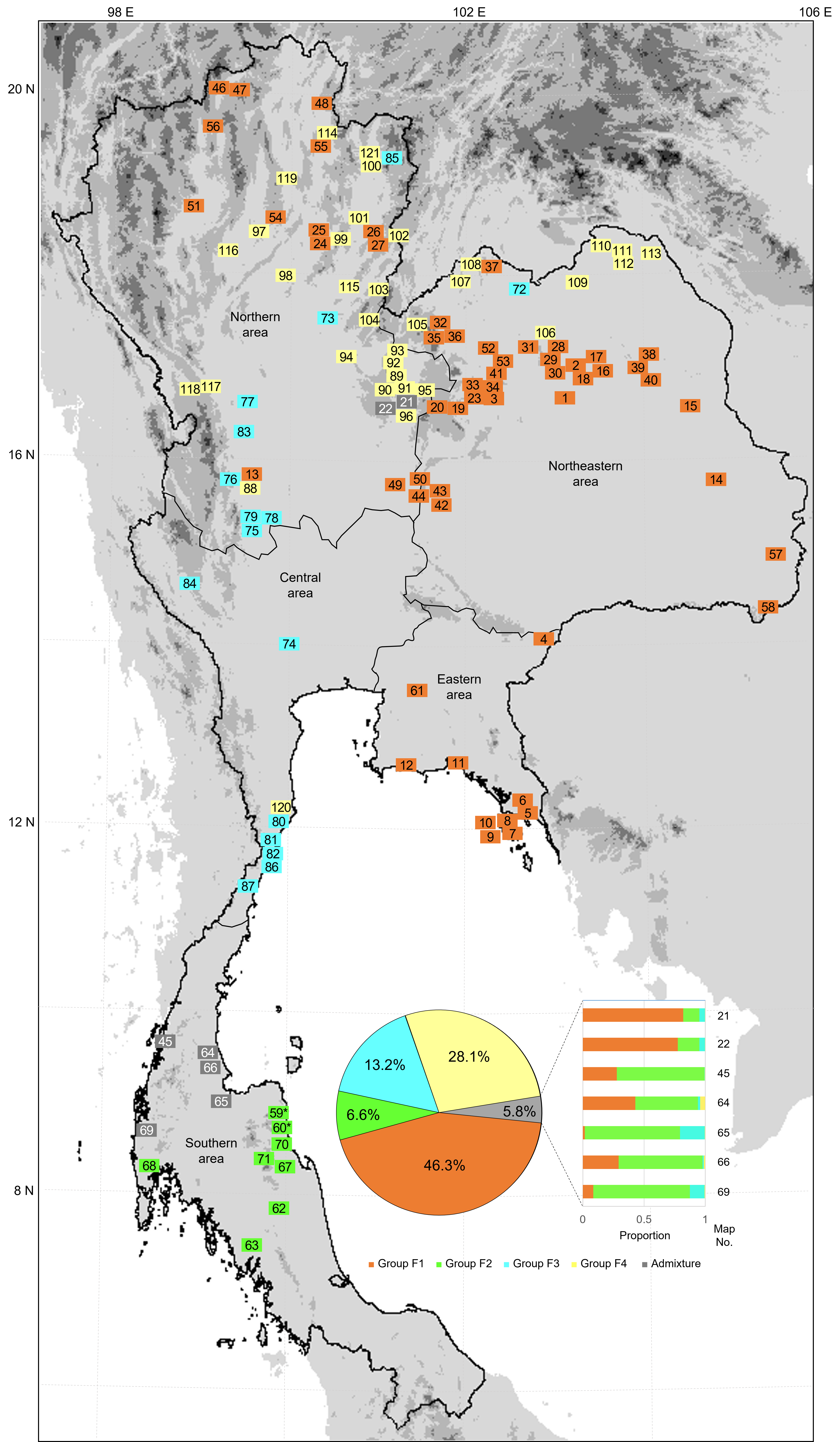

Supplement: Supplementary file 6 — Additional file 6: Figure S1. Geographic locations of 121 Erianthus accessions collected in Thailand, and a pie chart of the populations and a bar chart of ancestry proportion in the 7 admixtures. Colors correspond to those in Fig. 1 at K = 4. Admixture group is indicated in gray. Accession numbers are listed as map No. in Table S2. [file 12870_2021_3418_MOESM6_ESM.tif]
